# Quantitative thrombus characteristics on thin-slice computed tomography improve prediction of thrombus histopathology: results of the MR CLEAN Registry

**DOI:** 10.1007/s00330-022-08762-y

**Published:** 2022-04-30

**Authors:** Hajo Hund, Nikki Boodt, Nerea Arrarte Terreros, Aladdin Taha, Henk A. Marquering, Adriaan C. G. M. van Es, Reinoud P. H. Bokkers, Geert J. Lycklama à Nijeholt, Charles B.L.M. Majoie, Diederik W.J. Dippel, Hester F. Lingsma, Heleen M. M. van Beusekom, Aad van der Lugt, on behalf of the MR CLEAN Registry Investigators

**Affiliations:** 1 Department of Radiology, Haaglanden Medical Center, The Hague, the Netherlands; 2 Department of Radiology and Nuclear Medicine, Erasmus MC, University Medical Center Rotterdam, Doctor Molewaterplein 40, 3015 GD Rotterdam, the Netherlands; 3 Department of Neurology, Erasmus MC, University Medical Center Rotterdam, Rotterdam, the Netherlands; 4 Department of Cardiology, Erasmus MC, University Medical Center Rotterdam, Rotterdam, the Netherlands; 5 Department of Public Health, Erasmus MC, University Medical Center Rotterdam, Rotterdam, the Netherlands; 6 Department of Radiology and Nuclear Medicine, Amsterdam UMC, location AMC, Amsterdam, the Netherlands; 7 Department of Biomedical Engineering and Physics, Amsterdam UMC, location AMC, Amsterdam, the Netherlands; 8 Department of Radiology, Leiden University Medical Center, Leiden, the Netherlands; 9 Department of Radiology, University Medical Center Groningen, Groningen, the Netherlands

**Keywords:** Ischemic stroke, Computed tomography, Thrombus, Thrombectomy, Histopathology

## Abstract

**Objectives:**

Thrombus computed tomography (CT) characteristics might be used to assess histopathologic thrombus composition in patients treated with endovascular thrombectomy (EVT) for acute ischemic stroke (AIS). We aimed to assess the variability in thrombus composition that could be predicted with combined thrombus CT characteristics.

**Methods:**

Thrombi of patients enrolled in the MR CLEAN Registry between March 2014 and June 2016 were histologically analyzed with hematoxylin-eosin staining and quantified for percentages of red blood cells (RBCs) and fibrin/platelets. We estimated the association between general qualitative characteristics (hyperdense artery sign [HAS], occlusion location, clot burden score [CBS]) and thrombus composition with linear regression, and quantified RBC variability that could be explained with individual and combined characteristics with *R*^2^. For patients with available thin-slice (≤ 2.5 mm) imaging, we performed similar analyses for general and quantitative characteristics (HAS, occlusion location, CBS, [relative] thrombus density, thrombus length, perviousness, distance from ICA-terminus).

**Results:**

In 332 included patients, the presence of HAS (*aβ* 7.8 [95% CI 3.9–11.7]) and shift towards a more proximal occlusion location (*aβ* 3.9 [95% CI 0.6–7.1]) were independently associated with increased RBC and decreased fibrin/platelet content. With general characteristics, 12% of RBC variability could be explained; HAS was the strongest predictor. In 94 patients with available thin-slice imaging, 30% of RBC variability could be explained; thrombus density and thrombus length were the strongest predictors.

**Conclusions:**

Quantitative thrombus CT characteristics on thin-slice admission CT improve prediction of thrombus composition and might be used to further guide clinical decision-making in patients treated with EVT for AIS in the future.

**Key Points:**

*• With hyperdense artery sign and occlusion location, 12% of variability in thrombus RBC content can be explained.*

*• With hyperdense artery sign, occlusion location, and quantitative thrombus characteristics on thin-slice (≤ 2.5 mm) non-contrast CT and CTA, 30% of variability in thrombus RBC content can be explained.*

*• Absolute thrombus density and thrombus length were the strongest predictors for thrombus composition.*

## Introduction

Despite the evident clinical benefit of endovascular thrombectomy (EVT) for acute ischemic stroke (AIS), about 20–30% of thrombi are resistant to the current retrieval approaches [1]. The histological composition of the occluding thrombus, i.e., the percentages of red blood cells (RBCs), fibrin, and platelets in the thrombus, has been associated with angiographic and clinical outcomes of EVT. Fibrin/platelet-rich (RBC-poor) thrombi have been shown to be stiffer and are associated with more retrieval attempts, longer procedure times, lower revascularization rates, and less favorable clinical outcomes than their RBC-rich counterparts [2–6]. It has been suggested that if accurate information on thrombus composition could be acquired prior to EVT, the interventionalist would be provided with a sense of what to expect during the procedure [7]. Moreover, information on the thrombus’ histological composition may help guide treatment decisions, as scientific knowledge on thrombus-device interaction expands and thrombectomy devices specific for fibrin/platelet-rich thrombi are currently being developed [7–9].

Several thrombus characteristics can be assessed on non-contrast computed tomography (NCCT) and CT angiography (CTA), which are routinely conducted on hospital admission in patients with AIS. The presence of the hyperdense artery sign (HAS), based on visual identification of an artery with hyperdense attenuation, has been associated with RBC-rich (fibrin/platelet-poor) thrombi [5, 7, 10, 11]. The relationship of other general qualitative thrombus CT characteristics, such as occlusion location and clot burden score (CBS ranges from 0 to 10 and describes thrombus extent; a score of 10 is normal, and a score of 0 implies complete multisegment vessel occlusion [12]), with histological thrombus composition is not well-described.

As opposed to general qualitative thrombus CT characteristics, quantitative thrombus CT characteristics assessed on thin-slice (≤ 2.5 mm) CT [13–15] have the potential to provide a more accurate representation of thrombus subtype. Some studies have demonstrated the association of increased thrombus density (usually defined as the mean intraclot attenuation in Hounsfield units [HU] on NCCT) with increased RBC content of the thrombus [16–19], while others found no such association [3, 20–22]. Studies on the relationship between thrombus perviousness, which is a measure for thrombus permeability for contrast agent, and thrombus composition have reported heterogeneous results so far [21–24]. The relationship of thrombus length and measured distance from the internal carotid artery (ICA) terminus and thrombus composition has not been described previously.

Previous studies on the relationship of thrombus CT characteristics have been small in size and have mainly focused on one imaging characteristic at a time. Therefore, the value of a combination of thrombus CT characteristics to predict thrombus composition remains unknown. In this study, we aim to assess the relationship between various thrombus CT characteristics and thrombus composition, and to evaluate if thrombus composition can be predicted with individual and combined thrombus CT characteristics, in a large, multicenter registry of patients who underwent EVT for AIS.

## Materials and methods

### Setting and patients

We used data from the MR CLEAN (Multicenter Randomized Clinical Trial of Endovascular Treatment for Acute Ischemic Stroke in the Netherlands) Registry, which was a prospective observational study of all patients who underwent EVT for AIS in the Netherlands [25]. Enrollment started on 16 March 2014. All patients undergoing EVT (defined as at least entry into the angiography suite and receiving arterial puncture) for AIS, treated in one of the 16 centers performing EVT in the Netherlands, were registered. The central medical ethics committee of the Erasmus Medical Center Rotterdam, the Netherlands, approved the study protocol and granted permission to carry out the study as a registry (MEC-2014-235). Patients who met the following criteria were included in this study: age 18 years and older with a proximal intracranial vessel occlusion in the anterior circulation as demonstrated on CTA, and available thrombus for histological analysis. Data of patients treated until 15 June 2016 was collected and analyzed for this study.

### Histological thrombus composition

Histological analyses were performed according to previously described methods [26]. Directly after EVT, retrieved thrombi were fixated in 4% buffered formaldehyde. Thrombi were embedded in paraffin with the largest cross-sectional area available for sectioning, typically along the length of the thrombus. Thrombi retrieved with multiple passes were collected and analyzed together. Two 5-μm sections were acquired at representative depths, generally at 170 μm and 230 μm. Staessens and colleagues have shown that in large studies, one section per thrombus already provides accurate representation of thrombus composition [26]. Sections were stained with hematoxylin-eosin (H&E, HT110216, Sigma-Aldrich) and microscopical digital images were acquired with a slide scanner at 40× magnification (228 nm/pixel, 2.0 HT Nanozoomer, Hamamatsu Photonics). We quantitatively assessed RBC content and fibrin/platelet content, expressed as percentage of total thrombus section area, with the use of Orbit Image Analysis software (version 3.15, Idorsia Pharmaceuticals Ltd).

### Thrombus computed tomography characteristics

A central imaging core laboratory consisting of 21 observers (20 interventional neuroradiologists and 1 interventional neurologist, with at least 5–10 years of experience in diagnostic imaging and blinded for thrombus composition and all clinical information except for symptom side) assessed admission NCCT and CTA for general qualitative thrombus CT characteristics: presence of HAS, occlusion location, and CBS. Occlusion location (internal carotid artery [ICA], ICA-terminus, middle cerebral artery [proximal M1, distal M1, M2], anterior cerebral artery [A1, A2]) was based on the occluded segment on CTA.

In addition, quantitative thrombus CT characteristics were assessed in a subset of patients with available thin-slice (≤ 2.5 mm) NCCT and CTA imaging, acquired within 30 min from each other (available for 94 of 332 patients). Two neuroradiologists (7–8 years of experience in diagnostic imaging and blinded for thrombus composition) assessed absolute thrombus density, relative thrombus density, thrombus length, perviousness, and distance from the ICA-terminus to the thrombus (DT), according to previously described methods [13–15]. NCCT and CTA images were co-registered using rigid registration with Elastix [27]. Three spherical markers with a 1-mm radius were placed in the proximal, middle, and distal parts of the thrombus, on co-registered NCCT and CTA. Subsequently, three markers were symmetrically placed in the contralateral artery. Absolute thrombus density was defined as the mean density (in HU) of the three NCCT markers. Relative thrombus density was calculated by dividing the mean density of the thrombus by the mean density of the contralateral artery. Thrombus perviousness was estimated by subtracting the thrombus’ mean density on NCCT from its mean density on CTA. Additionally, one marker was placed at the proximal thrombus border and one marker at the distal thrombus border. Thrombus length was defined as the largest extension of contrast filling defect in the occluded vessel on CTA (in mm). Whenever the proximal or distal border of the thrombus could not be depicted on CTA, thrombus length was based on the hyperdense artery sign on the co-registered NCCT [28]. Lastly, DT represents the distance from the ICA-terminus to the beginning of the thrombus (in mm) and was manually measured on CTA using axial, coronal, and/or sagittal views [29]. For thrombi located in the supra-clinoid segment of the ICA but not extending into the ICA-terminus, DT was set to zero.

### Statistical analysis

For illustration, patient and baseline characteristics of the study population were tabulated according to tertiles of RBC content. The association of HAS, occlusion location, and CBS with thrombus composition (RBC content and fibrin/platelet content, expressed as percentage of the thrombus) was estimated with univariable and multivariable linear regression models and presented as (adjusted) coefficients (*aβ*) with 95% confidence intervals (CI). We performed two multivariable analyses. In the first multivariable model, we adjusted for the clinical variables age, sex, time from stroke onset to CT, and administration of intravenous alteplase. In the second model, we adjusted for thrombus CT characteristics. For the regression analyses, we treated occlusion location as a continuous variable, increasing from proximal to distal (ICA; ICA-T; proximal M1; distal M1; M2/A2). Additionally, in the subgroup with available thin-slice imaging, we assessed the association of HAS, occlusion location, CBS, absolute thrombus density, relative thrombus density, thrombus length, perviousness, and DT with thrombus composition, in a similar manner.

We estimated the variability in thrombus composition (RBC content and fibrin/platelet content) that was explained with thrombus CT characteristics with *R*^2^. *R*^2^ expresses the proportion of variance of a dependent variable that is explained by variables in a regression model. We reported *R*^2^ for increasingly extensive models, in which one CT characteristic at a time was added to the model. The order in which CT characteristics were added was based on our perceived clinical availability and easiness in assessment, starting with the characteristic that was easiest to acquire (HAS). Furthermore, we assessed the value of each individual thrombus CT characteristic, by assessing the uniquely added *R*^2^ to the full model (partial *R*^2^) for each thrombus CT characteristic. For the analyses, missing data were imputed using multiple imputation. Variables that were imputed are listed in the Data Appendix (Table 4 in the Data Appendix). STATA/SE 16.0 (StataCorp LLC) was used for all statistical analyses.

## Results

During the inclusion period, 1627 patients were registered. After excluding patients based on the prespecified criteria (*n* = 1295), 332 patients qualified for the assessment of general qualitative thrombus CT characteristics. Of 332 patients, 94 patients had an available thin-slice imaging for assessment of quantitative thrombus CT characteristics (Appendix Figure 4).

Of the 332 included patients, median age was 70 (interquartile range [IQR] 60–79) years, 177 (53%) were men, and median National Institutes of Health Stroke Scale on admission was 17 (IQR 13–20). The most common occlusion location was the proximal M1 (*n* = 99, 31%). HAS was present in 189 (60%) patients and median CBS was 6 (IQR 4–7). The majority of patients (*n* = 251, 76%) had received intravenous alteplase before EVT (Table 1). Median RBC content was 27% (IQR 16–42) and median fibrin/platelet content was 67% (IQR 53–78) (Appendix Figure 5). In the 94 patients with thin-slice imaging, median absolute thrombus density was 54.3 (IQR 46.4–59.6) HU, median relative thrombus density was 1.4 (IQR 1.2–1.6), median thrombus length was 15 (IQR 10–19) mm, median thrombus perviousness was 4.3 (IQR − 0.7 to 10.9) HU, and median DT was 5 (IQR 0–11) mm.

**Table 1:** Baseline, imaging, and workflow characteristics of patients in the current study, shown by tertiles of RBC content

	≤ 19.8% RBCs (*n* = 111)	19.9–36.7% RBCs (*n* = 111)	> 36.8% RBCs (*n* = 110)	Total (*n* = 332)
Age, year, median (IQR)	71 (58–80)	70 (63–79)	70 (59–76)	70 (60–79)
Men, *n* (%)	50 (45)	61 (55)	66 (60)	177 (53)
NIHSS, median (IQR)	17 (11–20)	18 (13–20)	17 (14–21)	17 (13–20)
Systolic blood pressure, mmHg, mean (SD)	147 (23)	149 (24)	151 (26)	149 (24)
Intravenous alteplase treatment, *n* (%)	85 (77)	85 (77)	81 (74)	251 (76)
Hyperdense artery sign, *n* (%)	48 (46)	63 (60)	78 (73)	189 (60)
Clot burden score, median (IQR)	6 (4–8)	6 (4–7)	5 (2–6)	6 (4–7)
*Medical history*				
Diabetes mellitus, *n* (%)	20 (18)	23 (21)	14 (13)	57 (17)
Hypertension, *n* (%)	58 (54)	61 (56)	59 (54)	178 (55)
Atrial fibrillation, *n* (%)	34 (31)	31 (28)	27 (25)	92 (28)
Ischemic stroke, *n* (%)	23 (21)	26 (24)	17 (16)	66 (20)
Peripheral artery disease, *n* (%)	17 (16)	9 (8)	17 (16)	43 (13)
Current smoking, *n* (%)	35 (32)	26 (23)	26 (24)	74 (22)
*Collateral grade*				
0, *n* (%)	3 (3)	10 (10)	14 (14)	27 (9)
1, *n* (%)	40 (39)	36 (36)	33 (32)	109 (36)
2, *n* (%)	35 (34)	42 (42)	36 (35)	113 (37)
3, *n* (%)	26 (25)	13 (13)	19 (19)	38 (19)
*Occlusion location subgroups*				
Intracranial ICA, *n* (%)	2 (2)	4 (4)	6 (6)	12 (4)
ICA-terminus, *n* (%)	19 (18)	27 (26)	44 (41)	90 (28)
Proximal M1, *n* (%)	34 (32)	29 (28)	36 (34)	99 (31)
Distal M1, *n* (%)	39 (36)	34 (33)	18 (17)	91 (29)
M2, *n* (%)	13 (12)	9 (9)	3 (3)	25 (8)
A1/A2, *n* (%)	1 (1)	0 (0)	0 (0)	1 (0)
*Workflow*				
Time from stroke onset to CT, min, median (IQR)	72 (60–110)	65 (50–113)	69 (53–115)	69 (53–114)
Time from stroke onset to groin puncture, min, median (IQR)	200 (155–260)	205 (155–266)	209 (166–260)	205 (160–260)

*IQR* indicates interquartile range, *NIHSS* National Institutes of Health Stroke Scale

*SD* standard deviation, *ICA* internal carotid artery, *M1* and *M2* middle cerebral artery, *A1* and *A2* anterior cerebral artery, *CT* computed tomography

### Association of thrombus CT characteristics with thrombus composition

**Fig. 1:**
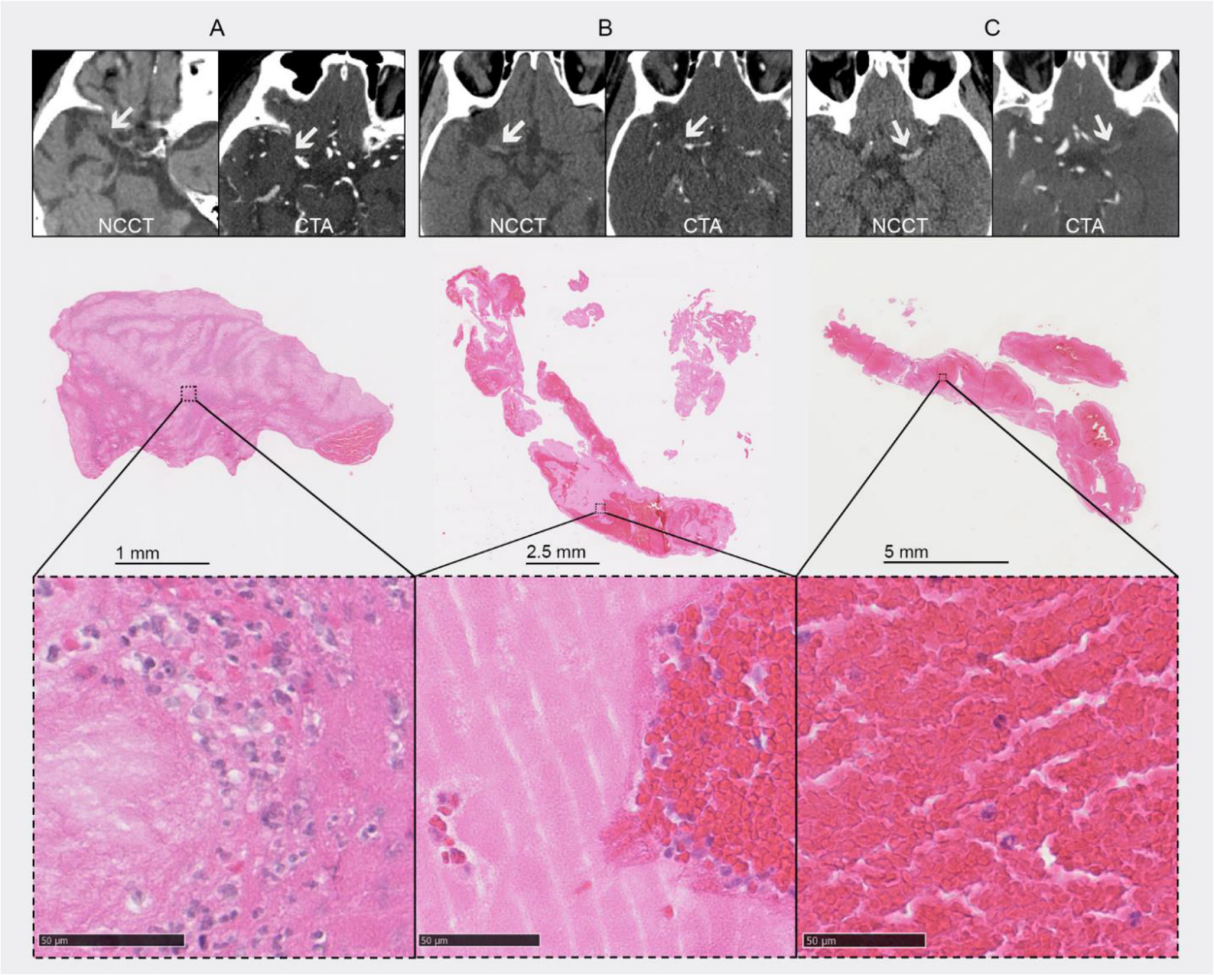
Thrombi on computed tomography and the corresponding histological images, shown according to increasing red blood cell (RBC) content. From left to right (mean absolute thrombus density, RBC content): 32.4 HU, 4.3% (**A**); 54.0 HU, 25.6% (**B**); and 61.5 HU, 75.4% (**C**)

**Fig. 2:**
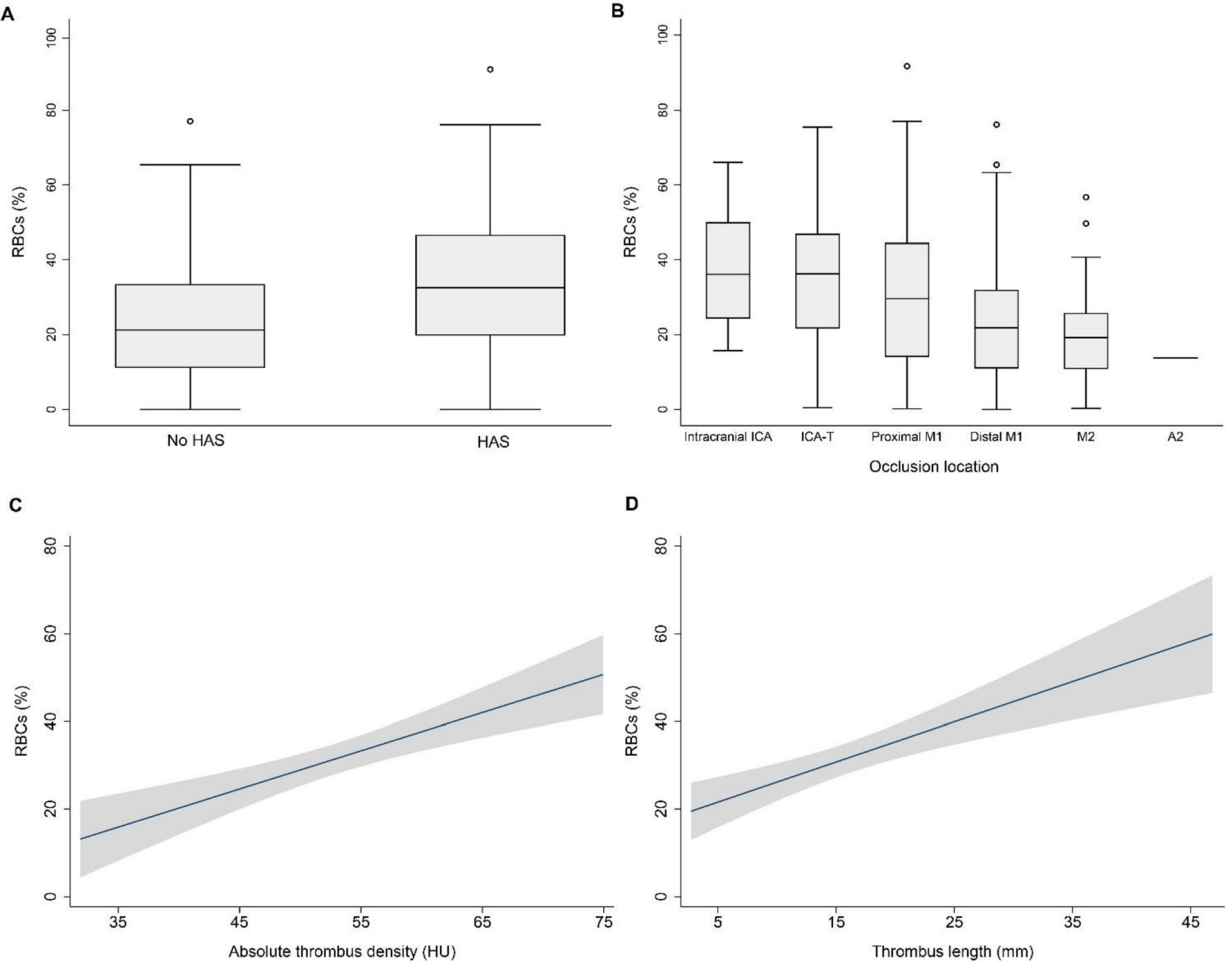
Boxplots of the relationship of hyperdense artery sign (HAS) and occlusion location with red blood cell (RBC) content (**A** and **B**, *n* = 332) and fitted curves with 95% confidence intervals for the relationship of absolute thrombus density and thrombus length with RBC content (**C** and **D**, *n* = 94)

Thrombus CT characteristics were associated with thrombus composition (Figs. 1 and 2). In all patients (*n* = 332), the presence of HAS (*β* 9.8 [95% CI 5.8 to 13.7]), shift towards a more proximal occlusion location (*β* − 5.5 [95% CI − 7.5 to − 3.5]), and lower CBS (*β* − .9 [95% CI − 2.7 to − 1.1]) were associated with increased RBC content (Table 2) and with decreased fibrin/platelet content (Appendix Table 5) in the univariable analyses. Adjustment for clinical characteristics did not change these associations. When we adjusted for thrombus CT characteristics, HAS and occlusion location were independently associated with RBC content and fibrin/platelet content, while CBS was not.

**Table 2:** Univariable and multivariable linear regression analyses for the relationship between thrombus CT characteristics and thrombus RBC content, in all included patients (*n* = 332)

	Unadjusted	Adjusted for clinical characteristics*	Adjusted for thrombus CT characteristics†
Hyperdense artery sign	9.8 (5.8 to 13.7)	9.8 (5.8 to 13.7)	7.8 (3.9 to 11.7)
Occlusion location	− 5.5 (− 7.5 to − 3.5)	− 5.5 (− 7.6 to -3.5)	− 3.9 (− 7.1 to − 0.6)
Clot burden score	− 1.9 (− 2.7 to − 1.1)	− 1.9 (− 2.7 to − 1.1)	− 0.4 (− 1.7 to 0.9)

Regression coefficients are shown with 95% confidence intervals. All analyses were done with RBC content as a continuous variable, expressed as percentage of the thrombus

*Age, sex, time from stroke onset to CT, and administration of IV alteplase

†Hyperdense artery sign, occlusion location, and clot burden score

In the subgroup with available thin-slice imaging (*n* = 94), absolute thrombus density (*β* 0.9 [95% CI 0.5 to 1.3]), thrombus length (*β* 0.9 [95% CI 0.5 to 1.4]), decreased thrombus perviousness (*β* − 0.4 [95% CI − 0.6 to − 0.1]), and decreased DT (*β* − 0.6 [95% CI − 1.1 to − 0.0]) were associated with increased RBC content (Table 3) and with decreased fibrin/platelet content (Appendix Table 6) in the univariable analyses. These associations remained unchanged when we adjusted for the clinical variables. When we adjusted for thrombus CT characteristics, absolute thrombus density and thrombus length were independently associated with thrombus composition, while all other characteristics were not (Table 3).

**Table 3:** Univariable and multivariable linear regression analyses for the relationship between thrombus CT characteristics and thrombus RBC content, in patients with available thin-slice CT imaging (*n* = 94)

	Unadjusted	Adjusted for clinical characteristics*	Adjusted for thrombus CT characteristics†
Hyperdense artery sign	5.5 (− 2.4 to 13.3)	5.7 (− 2.1 to 13.5)	− 3.7 (− 11.7 to 4.2)
Occlusion location	− 4.4 (− 8.4 to − 0.4)	− 4.0 (− 8.1 to 0.1)	− 4.1 (− 13.5 to 5.4)
Clot burden score	− 1.7 (− 3.3 to − 0.1)	− 1.4 (− 3.0 to 0.2)	1.0 (− 2.2 to 4.1)
Absolute thrombus density (HU)	0.9 (0.5 to 1.3)	0.8 (0.5 to 1.2)	0.8 (0.3 to 1.4)
Thrombus length (mm)	0.9 (0.5 to 1.3)	0.8 (0.4 to 1.3)	0.8 (0.2 to 1.4)
Relative thrombus density (HU)	13.1 (− 0.8 to 27.1)	14.2 (0.4 to 28.0)	− 10.4 (− 26.8 to 6.1)
Perviousness (HU)	− 0.4 (− 0.6 to − 0.1)	− 0.4 (− 0.6 to − 0.1)	− 0.2 (− 0.4 to 0.0)
DT (mm)	− 0.6 (− 1.1 to − 0.0)	− 0.5 (− 1.0 to 0.1)	0.6 (− 0.3 to 1.5)

Regression coefficients are shown with 95% confidence intervals. All analyses were done with RBC content as a continuous variable, expressed as percentage of the thrombus. HU indicates Hounsfield units and DT, distance from internal carotid artery terminus to thrombus

*Age, sex, time from stroke onset to CT, and administration of IV alteplase

†Hyperdense artery sign, occlusion location, clot burden score, absolute thrombus density, thrombus length, relative thrombus density, perviousness and distance from internal carotid artery terminus to the thrombus

### Explained variability in thrombus composition

**Fig. 3:**
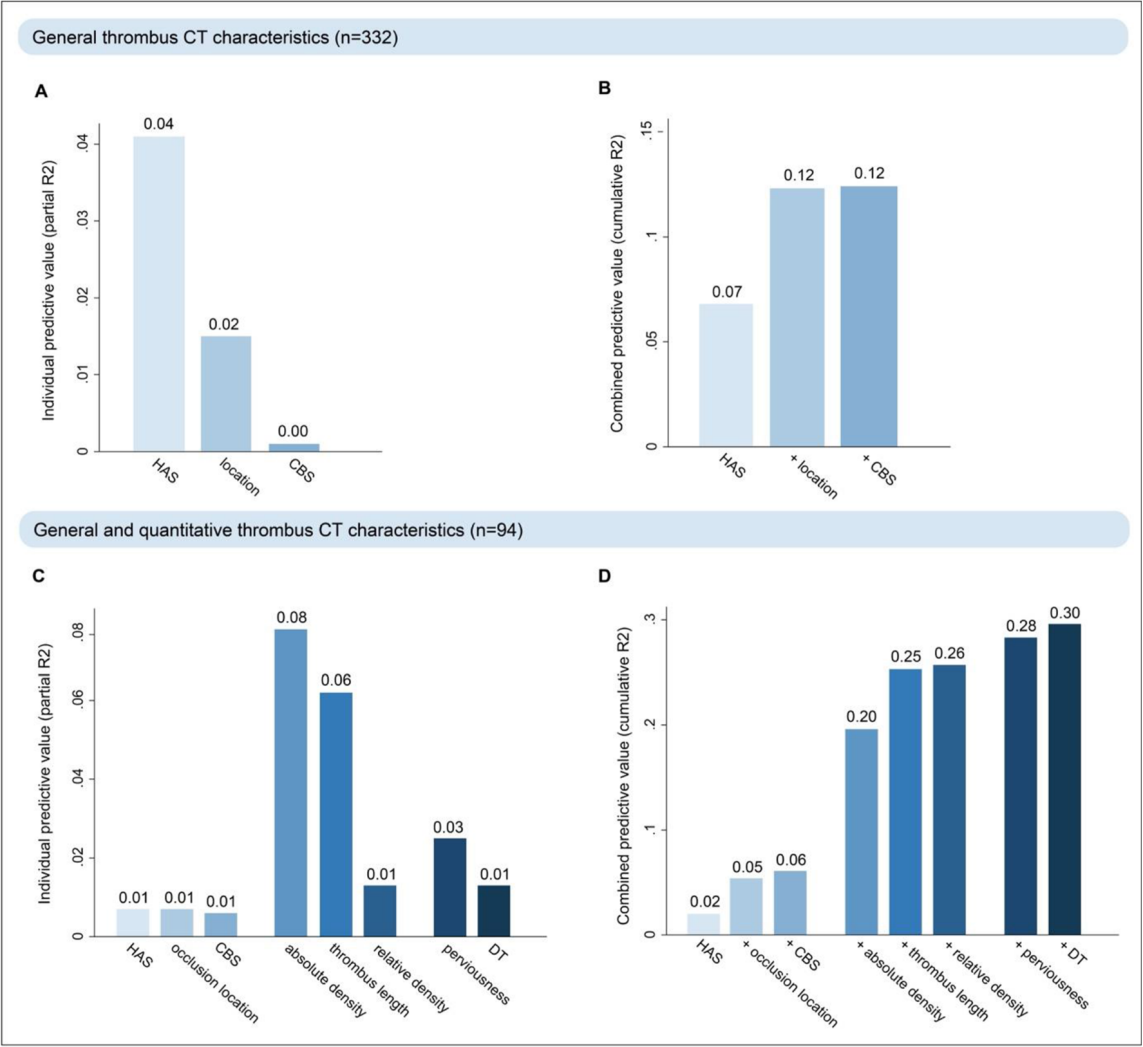
Explained variability in thrombus red blood cell (RBC) content by thrombus CT characteristics is shown. Of the general qualitative characteristics (hyperdense artery sign [HAS], occlusion location and clot burden score [CBS]), HAS was the strongest predictor (**A**) and 12% of RBC variability could be explained with these characteristics combined (**B**). In patients with available thin-slice imaging, thrombus density and thrombus length were the strongest predictors (**C**) and 30% of RBC variability could be explained with all thrombus CT characteristics combined (**D**)

Of the general thrombus CT characteristics (HAS, occlusion location and CBS, *n* = 332), HAS was the strongest predictor for RBC content, followed by occlusion location (Fig. 3a). A model including HAS and occlusion location explained 12% of the variability in RBC content of the thrombus (Fig. 3b). Adding CBS did not improve the performance of this model.

Of all thrombus CT characteristics (HAS, occlusion location, CBS, [relative] thrombus density, thrombus length, perviousness, and DT, in the subset of patients with thin-slice imaging, *n* = 94), absolute thrombus density and thrombus length were the strongest predictors for RBC content (Fig. 3c). The most extensive model, including all thrombus CT characteristics, explained 30% of the variability in RBC content of the thrombus (Fig. 3d).

## Discussion

In this study, we have shown that thrombus CT characteristics are associated with the histological composition of the occluding thrombus in patients treated with EVT for AIS. Additionally, we have demonstrated that about 30% of the variability in thrombus composition can be explained with a combination of thrombus CT characteristics derived from admission thin-slice NCCT and CTA.

In line with previous literature, we found that the presence of HAS was associated with increased RBC content and decreased fibrin/platelet content of the thrombus [5, 7]. We also observed a clear relationship between increased absolute thrombus density and increased RBC content and decreased fibrin/platelet content, which is in line with most studies on this topic [16–19], but not with some others [3, 20–22]. Noteworthy, the majority of studies that found a relationship between thrombus density and thrombus composition used imaging with 5-mm slice thickness and one region of interest for the assessment of thrombus density, while the value of thin-slice imaging and multiple regions of interest has been shown [13]. Interestingly, we did not observe a relationship between relative thrombus density and thrombus composition, which is in contrast to earlier findings [17, 22]. Relative thrombus density is calculated from regions of interest in the thrombus as well as in the contralateral artery, and has been shown to have higher interobserver variability than absolute thrombus density [13], which could be an explanation for our conflicting findings. We observed a weak association between decreased thrombus perviousness and increased RBC content, which is in line with previous contrasting findings regarding the relationship between thrombus perviousness and thrombus histology [21–24]. Increased thrombus length on CT was associated with increased RBC content and decreased fibrin/platelet content. To our knowledge, this relationship has not been described in previous literature. However, in line with our findings, a more proximal occlusion location has previously been associated with increased RBC content [30]. Since smaller thrombi are more likely to travel further into the intracranial circulation [31], this relationship might be explained due to collinearity between thrombus length and location.

The value of combined thrombus CT characteristics for the prediction of histological thrombus composition is not well-described. In this study, we have shown that quantitative thrombus CT characteristics substantially improved prediction of thrombus composition: With a combination of general and quantitative thrombus CT characteristics, 30% of the variability in thrombus composition could be explained, as opposed to 12% when only general thrombus CT characteristics were available. Future studies should aim to confirm these findings.

### Thrombus CT characteristics as a potential guide for EVT approach

Recent in vitro studies have shown that with stent retrievers, better results are achieved for soft, RBC-rich thrombi than for stiffer, fibrin/platelet-rich thrombi [3, 7]. Therefore, it has been suggested that selection of first-line device (i.e., aspiration versus stent retriever thrombectomy) might be based on thrombus composition in the future. Moreover, research on next-generation EVT devices currently focuses on specific designs for fibrin-rich thrombi. In the current study, we have shown that a substantial part of thrombus composition can be explained with thrombus CT characteristics. Future studies could focus on whether certain thrombectomy techniques or devices tailored to thrombus CT characteristics, such as density and length, result in better outcomes, to eventually reach complete reperfusion in all patients.

### Limitations

This study has limitations. First, a large proportion of patients who underwent EVT was not included in the study, because the thrombus was not kept for histological processing at the participating center or no thrombus was retrieved during the intervention. Unfortunately, we did not keep track of in which cases a retrieved thrombus was not sent in for histology, which is a consequence of a multicenter registry of daily clinical practice. Furthermore, studies have shown that RBC-rich thrombi, or parts of the thrombus that are RBC-rich, are more likely to be successfully retrieved with EVT. Therefore, fibrin/platelet-rich thrombi might have been underrepresented in our study. Moreover, for the quantification of histological thrombus components, it is assumed that thrombus composition is homogeneous throughout the thrombus, while in reality, thrombi are highly heterogeneous. However, Staessens and colleagues have shown that in large studies, one section per thrombus already provides accurate representation of total thrombus composition. Lastly, fibrin and platelets cannot be easily distinguished from each other on H&E staining. While most previous studies on histological thrombus composition quantified components in the same manner as we did, future studies should assess fibrin and platelets separately.

## Conclusions

Quantitative thrombus CT characteristics derived from thin-slice (≤ 2.5 mm) admission NCCT and CTA imaging improve prediction of thrombus composition and might be used to guide clinical decision-making in patients treated with EVT for AIS in the future.

## Figures and Tables

**Fig. 4:**
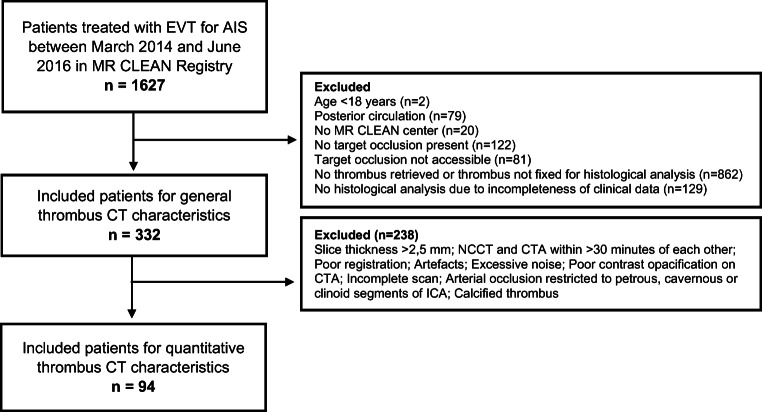
Flowchart of patient inclusion. EVT indicates endovascular thrombectomy; AIS, acute ischemic stroke; NCCT, non-contrast computed tomography; CTA, computed tomography angiography and ICA, internal carotid artery

**Fig. 5:**
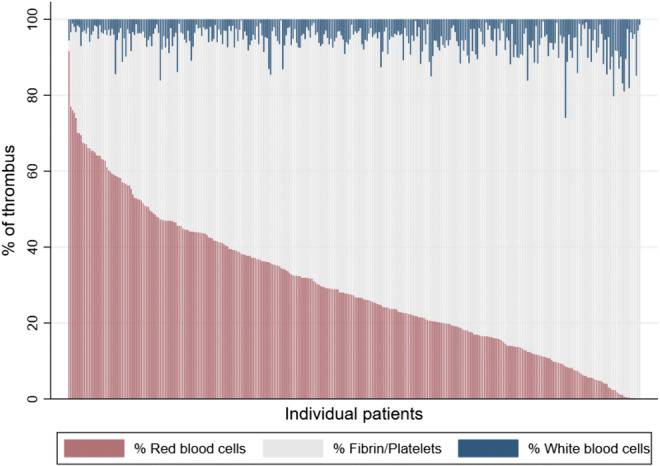
Histological thrombus composition of the 332 included patients

## Appendix

### MR CLEAN Registry Investigators – group authors

#### Executive committee

Diederik W.J. Dippel^1^;Aad van der Lugt^2^;Charles B.L.M. Majoie^3^;Yvo B.W.E.M. Roos^4^;Robert J. van Oostenbrugge^5^;Wim H. van Zwam^6^;Jelis Boiten^14^;Jan Albert Vos^8^

#### Study coordinators

Ivo G.H. Jansen^3^;Maxim J.H.L. Mulder^1,2^;Robert- Jan B. Goldhoorn^5,6^;Kars C.J. Compagne^2^;Manon Kappelhof^3^;Josje Brouwer^4^;Sanne J. den Hartog^1,2,40^;Wouter H. Hinsenveld ^5,6^;

#### Local principal investigators

Diederik W.J. Dippel^1^;Bob Roozenbeek^1^;Aad van der Lugt^2^;Adriaan C.G.M. van Es^2^;Charles B.L.M. Majoie^3^;Yvo B.W.E.M. Roos^4^;Bart J. Emmer^3^;Jonathan M. Coutinho^4^;Wouter J. Schonewille^7^;Jan Albert Vos^8^; Marieke J.H. Wermer^9^;Marianne A.A. van Walderveen^10^;Julie Staals^5^;Robert J. van Oostenbrugge^5^;Wim H. van Zwam^6^;Jeannette Hofmeijer^11^;Jasper M. Martens^12^;Geert J. Lycklama à Nijeholt^13^;Jelis Boiten^14^;Sebastiaan F. de Bruijn^15^;Lukas C. van Dijk^16^;H. Bart van der Worp^17^;Rob H. Lo^18^;Ewoud J. van Dijk^19^;Hieronymus D. Boogaarts^20^;J. de Vries^22^;Paul L.M. de Kort^21^; Julia van Tuijl^21^ ; Jo P. Peluso^26^;Puck Fransen^22^;Jan S.P. van den Berg^22^;Boudewijn A.A.M. van Hasselt^23^;Leo A.M. Aerden^24^;René J. Dallinga^25^;Maarten Uyttenboogaart^28^;Omid Eschgi^29^;Reinoud P.H. Bokkers^29^;Tobien H.C.M.L. Schreuder^30^;Roel J.J. Heijboer^31^;Koos Keizer^32^;Lonneke S.F. Yo^33^;Heleen M. den Hertog^22^;Tomas Bulut^35^; Paul J.A.M. Brouwers^34^

#### Imaging assessment committee

Charles B.L.M. Majoie^3^(chair);Wim H. van Zwam^6^;Aad van der Lugt^2^;Geert J. Lycklama à Nijeholt^13^;Marianne A.A. van Walderveen^10^;Marieke E.S. Sprengers^3^;Sjoerd F.M. Jenniskens^27^;René van den Berg^3^;Albert J. Yoo^38^;Ludo F.M. Beenen^3^;Alida A. Postma^6^;Stefan D. Roosendaal^3^;Bas F.W. van der Kallen^13^;Ido R. van den Wijngaard^13^;Adriaan C.G.M. van Es^2^;Bart J. Emmer^,3^;Jasper M. Martens^12^; Lonneke S.F. Yo^33^;Jan Albert Vos^8^; Joost Bot^36^, Pieter-Jan van Doormaal^2^; Anton Meijer^27^;Elyas Ghariq^13^; Reinoud P.H. Bokkers^29^;Marc P. van Proosdij^37^;G. Menno Krietemeijer^33^;Jo P. Peluso^26^;Hieronymus D. Boogaarts^20^;Rob Lo^18^;Wouter Dinkelaar^2^Auke P.A. Appelman^29^;Bas Hammer^16^;Sjoert Pegge^27^;Anouk van der Hoorn^29^;Saman Vinke^20^.

#### Writing committee

Diederik W.J. Dippel^1^(chair);Aad van der Lugt^2^;Charles B.L.M. Majoie^3^;Yvo B.W.E.M. Roos^4^;Robert J. van Oostenbrugge^5^;Wim H. van Zwam^6^;Geert J. Lycklama à Nijeholt^13^;Jelis Boiten^14^;Jan Albert Vos^8^;Wouter J. Schonewille^7^;Jeannette Hofmeijer^11^;Jasper M. Martens^12^;H. Bart van der Worp^17^;Rob H. Lo^18^

#### Adverse event committee

Robert J. van Oostenbrugge^5^(chair);Jeannette Hofmeijer^11^;H. Zwenneke Flach^23^

#### Trial methodologist

Hester F. Lingsma^40^

#### Research nurses / local trial coordinators

Naziha el Ghannouti^1^;Martin Sterrenberg^1^;Wilma Pellikaan^7^;Rita Sprengers^4^;Marjan Elfrink^11^;Michelle Simons^11^;Marjolein Vossers^12^;Joke de Meris^14^;Tamara Vermeulen^14^;Annet Geerlings^19^;Gina van Vemde^22^;Tiny Simons^30^;Gert Messchendorp^28^;Nynke Nicolaij^28^;Hester Bongenaar^32^;Karin Bodde^24^;Sandra Kleijn^34^;Jasmijn Lodico^34^; Hanneke Droste^34^;Maureen Wollaert^5^;Sabrina Verheesen^5^;D. Jeurrissen^5^;Erna Bos^9^;Yvonne Drabbe^15^;Michelle Sandiman^15^;Nicoline Aaldering^11^;Berber Zweedijk^17^;Jocova Vervoort^21^;Eva Ponjee^22^;Sharon Romviel^19^;Karin Kanselaar^19^;Denn Barning^10^.

#### PhD / Medical students:

Esmee Venema^40^; Vicky Chalos^1,40^; Ralph R. Geuskens^3^; Tim van Straaten^19^;Saliha Ergezen^1^; Roger R.M. Harmsma^1^; Daan Muijres^1^; Anouk de Jong^1^;Olvert A. Berkhemer^1,3,6^;Anna M.M. Boers^3,39^; J. Huguet^3^;P.F.C. Groot^3^;Marieke A. Mens^3^;Katinka R. van Kranendonk^3^;Kilian M. Treurniet^3^;Manon L. Tolhuisen^3,39^;Heitor Alves^3^;Annick J. Weterings^3^,Eleonora L.F. Kirkels^3^,Eva J.H.F. Voogd^11^;Lieve M. Schupp^3^;Sabine L. Collette^28,29^;Adrien E.D. Groot^4^;Natalie E. LeCouffe^4^;Praneeta R. Konduri^39^;Haryadi Prasetya^39^;Nerea Arrarte-Terreros^39^;Lucas A. Ramos^39^.

#### List of affiliations

Department of Neurology^1^, Radiology^2^, Public Health^40^, Erasmus MC University Medical Center;

Department of Radiology and Nuclear Medicine^3^, Neurology^4^, Biomedical Engineering & Physics^39^, Amsterdam UMC, University of Amsterdam, Amsterdam;

Department of Neurology^5^, Radiology^6^, Maastricht University Medical Center and Cardiovascular Research Institute Maastricht (CARIM);

Department of Neurology^7^, Radiology^8^, Sint Antonius Hospital, Nieuwegein;

Department of Neurology^9^, Radiology^10^, Leiden University Medical Center;

Department of Neurology^11^, Radiology^12^, Rijnstate Hospital, Arnhem;

Department of Radiology^13^, Neurology^14^, Haaglanden MC, the Hague;

Department of Neurology^15^, Radiology^16^, HAGA Hospital, the Hague;

Department of Neurology^17^, Radiology^18^, University Medical Center Utrecht;

Department of Neurology^19^, Neurosurgery^20^, Radiology^27^, Radboud University Medical Center, Nijmegen;

Department of Neurology^21^, Radiology^26^, Elisabeth-TweeSteden ziekenhuis, Tilburg;

Department of Neurology^22^, Radiology^23^, Isala Klinieken, Zwolle;

Department of Neurology^24^, Radiology^25^, Reinier de Graaf Gasthuis, Delft;

Department of Neurology^28^, Radiology^29^, University Medical Center Groningen;

Department of Neurology^30^, Radiology^31^, Atrium Medical Center, Heerlen;

Department of Neurology^32^, Radiology^33^, Catharina Hospital, Eindhoven;

Department of Neurology^34^, Radiology^35^, Medisch Spectrum Twente, Enschede;

Department of Radiology^36^, Amsterdam UMC, Vrije Universiteit van Amsterdam, Amsterdam;Department of Radiology^37^, Noordwest Ziekenhuisgroep, Alkmaar;

Department of Radiology^38^, Texas Stroke Institute, Texas, United States of America.

**Table 4:** Variables used for multiple imputation and theirfrequency of missing values

Variable	Frequency of missing values, n (%)
Age	0/332 (0)
Sex	0/332 (0)
Baseline NIHSS	1/332 (0)
Time from stroke onset to imaging	109/332 (33)
Intravenous alteplase	0/332 (0)
Occlusion location	14/332 (4)
Presence of hyperdense artery sign	15/332 (5)
Clot Burden Score	48/332 (15)

**Table 5:** Univariable and multivariable linear regression analyses for the relationship between thrombus CT characteristics and thrombus fibrin/platelet-content, in all included patients (*n* = 332)

	Unadjusted	Adjusted for clinical characteristics^a^	Adjusted for thrombus CT characteristics^b^
Hyperdense artery sign	-9.6 (-13.4 to -5.8)	-9.6 (-13.4 to -5.9)	-7.8 (-11.5 to -4.0)
Occlusion location	5.2 (3.2 to 7.1)	5.2 (3.3 to 7.2)	3.7 (0.5 to 6.8)
Clot Burden Score	1.8 (1.0 to 2.6)	1.8 (1.0 to 2.6)	0.3 (-0.9 to 1.6)

All analyses were done with fibrin/platelet content as a continuous variable, expressed as % of the thrombus. Regression coefficients are shown with 95% confidence intervals

^a^Age, sex, time from stroke onset to CT and administration of IV alteplase

^b^Hyperdense artery sign, occlusion location and Clot Burden Score

**Table 6:** Univariable and multivariable linear regression analyses for therelationship between thrombus CT characteristics and thrombus fibrin/platelet-content,in patients with available thin-slice CT imaging (*n* = 94)

	Unadjusted	Adjusted for clinical characteristics^a^	Adjusted for thrombus CT characteristics^b^
Hyperdense artery sign	-5.3 (-12.9 to 2.2)	-5.5 (-13.0 to 1.9)	3.2 (-4.5 to 10.9)
Occlusion location	4.2 (0.3 to 8.0)	3.8 (-0.1 to 7.8)	3.8 (-5.4 to 12.9)
Clot Burden Score	1.6 (0.1 to 3.1)	1.3 (-0.2 to 2.8)	-0.9 (-3.9 to 2.1)
Absolute thrombus density (HU)	-0.8 (-1.2 to -0.5)	-0.8 (-1.2 to -0.4)	-0.8 (-1.3 to -0.3)
Thrombus length (mm)	-0.8 (-1.2 to -0.4)	-0.7 (-1.2 to -0.3)	-0.7 (-1.3 to -0.1)
Relative thrombus density (HU)	-11.7 (-25.0 to 1.7)	-12.8 (-26.1 to 0.5)	10.3 (-5.7 to 26.3)
Perviousness (HU)	0.3 (0.1 to 0.6)	0.3 (0.1 to 0.6)	0.2 (-0.0 to 0.4)
DT (mm)	0.6 (0.0 to 1.1)	0.5 (-0.1 to 1.0)	-0.5 (-1.4 to 0.4)

All analyses were done with fibrin/platelet content as a continuous variable, expressed as % of the thrombus. Regression coefficients are shown with 95% confidence intervals. HU indicates Hounsfield Units and DT, distance from internal carotid artery terminus to thrombus

^a^Age, sex, time from stroke onset to CT and administration of IV alteplase

^b^Hyperdense artery sign, occlusion location, Clot Burden Score, absolute thrombus density, thrombus length, relative thrombus density, perviousness and distance from internal carotid artery terminus to thrombus

## Abbreviations

CBS: Clot burden score
CI: Confidence interval
IQR: Interquartile range
DT: Distance from ICA-terminus to thrombus in millimeters
HAS: Hyperdense artery sign
ICA: Internal carotid artery
*R*^2^: Statistical measure: proportion of the variance for a dependent variable that is explained by variables in a regression model
RBC: Red blood cell

## Declarations

### Guarantor

The scientific guarantor of this publication is prof. Aad van der Lugt, Department of Radiology and Nuclear Medicine, Erasmus University Medical Center, Rotterdam, the Netherlands.

### Statistics and biometry

One of the authors has significant statistical expertise.

### Methodology

• prospective

• observational

• multicenter study
